# Contribution of improved quality of early essential newborn care to neonatal mortality decline in Cambodia, 2010–2022: a retrospective observational study

**DOI:** 10.1136/bmjoq-2025-003920

**Published:** 2026-09-01

**Authors:** Sidonn Krang, Tung Rathavy, John Charles Scott Murray, Rattana Kim, Sano Phal, Priya Mannava, Kannitha Cheang, Y Meng Chhour, Howard Lawrence Sobel

**Affiliations:** 1 Department of Communicable Diseases Control, Royal Government of Cambodia Ministry of Health, Phnom Penh, Cambodia; 2 National Maternal and Child Health Center, Royal Government of Cambodia Ministry of Health, Phnom Penh, Cambodia; 3 World Health Organization Regional Office for the Western Pacific, Manila, Philippines; 4 WHO Representative Office Cambodia, Phnom Penh, Cambodia; 5 Under-Secretary of State for Health, Royal Government of Cambodia Ministry of Health, Phnom Penh, Cambodia

**Keywords:** EPIDEMIOLOGY, Paediatrics, Clinical practice guidelines

## Abstract

**Introduction:**

Between 2005 and 2010, reductions in newborn mortality fell behind those for maternal and under-five child mortality in Cambodia. To improve newborn outcomes, we developed and scaled-up early essential newborn care (EENC) nationally using a new clinical coaching and data-based quality improvement approach. This study aimed to determine EENC’s contribution to Cambodia’s observed neonatal mortality decline between 2010 and 2022.

**Methods:**

Standard indicators in the EENC monitoring and evaluation framework were calculated from programme records, nationally representative population-based surveys and facility-based assessments using standardised methods. The Lives Saved Tool (LiST) was used to model newborn lives saved 2010–2022 using facility estimates of care quality.

**Results:**

Newborn mortality declined from 27 to 8/1000 live births (70.4%) comparing 2010 and 2022. The average annual rate of newborn mortality decline accelerated by 33% after EENC introduction in 2012 compared with the previous 10 years, with greatest declines among high-risk groups (poorest, rural, uneducated and with low birth interval). Between 2010 and 2022, EENC was integrated into national and subnational policies, standards and implementation plans and introduced to 87.6% of birth facilities. The average birth practice score rose to 72.9% (95% CI 59.0% to 83.4%) in 2022, from 6% (95% CI 1.7% to 20.1%) in 2011. Significant improvements were seen in application of clean birth environments, immediate drying and stimulation, thermal protection (immediate and prolonged skin to skin contact), delayed cord clamping and clean cord care. LiST modelling estimated that EENC interventions averted 21 704 Cambodian newborn deaths (range 17 694–26 450) during 2010–2022 and were responsible for 75.3% (range 69.5%–80.5%) of the observed national mortality reduction.

**Conclusion:**

A new approach to EENC programme implementation led to sustained country-wide improvements in evidence-based practices around birth. Improved care quality was associated with 69.5–80.5% of the observed neonatal mortality decline in Cambodia between 2010 and 2022.

**WHAT IS ALREADY KNOWN ON THIS TOPIC**

- Simple evidence-based interventions delivered at birth or in the early newborn period have been shown to prevent or treat the most important causes of newborn morbidity and mortality.

**WHAT THIS STUDY ADDS**

- Cambodia introduced and scaled-up a new approach to improving the quality of early essential newborn care (EENC) between 2012 and 2022, with high national EENC coaching coverage and sustained improvements in evidence-based practices around birth.
- Newborn mortality declined from 27 to 8/1000 live births during 2010–2022, with the average annual rate of newborn mortality decline accelerating by 33% after EENC introduction.
- Lives Saved Tool modelling estimated that EENC interventions averted 21 704 newborn deaths during 2010–2022 (range 17 694–26 450) and were responsible for 75.3% (range 69.5–80.5%) of the observed national mortality decline.

**HOW THIS STUDY MIGHT AFFECT RESEARCH, PRACTICE OR POLICY**

- Sustained improvements in the quality of early newborn care are possible using facility-based on-the-job coaching driven by multidisciplinary hospital-teams; and by periodic collection and immediate use of practice data for tracking progress and making programme decisions.

## Introduction

 Cambodia achieved maternal and under-five mortality reductions exceeding 80% between 1990 and 2015, making it one of the seven countries globally that met Millennium Development Goal (MDG) 4: Reduction of under-five mortality rate by two-thirds and 5A: Reduction of maternal deaths by at least 75%.^1 2^ However, neonatal mortality declined more slowly after 1990, with the proportion of under-five deaths in the neonatal period rising from 35% to 51.5% through 2014.^3^ While skilled attended births increased by 60% and maternal mortality declined from 472 to 206 per 100 000 live births between 2005 and 2010, newborn mortality remained unchanged at 28 and 27 per 1000 live births^.4^

To address this challenge, the Ministry of Health in Cambodia initiated a strategic shift towards improving the quality of early newborn care. This shift was built on recognition that up to two-thirds of neonatal deaths occur in the first 3 days of life, mainly from complications related to prematurity, birth asphyxia and infection; and that health workers in Cambodia and elsewhere in Asia often used outdated and harmful clinical practices during and after delivery, increasing the risk for newborn morbidity and mortality.^5 6^ Further, intensive investment in improving access to and availability of birth care and rapidly rising rates of skilled birth attendance in Cambodia made improving the quality of newborn care around birth a priority.^7^

In 2011, the Ministry of Health partnered with the WHO to develop, test and scale-up early essential newborn care (EENC), a package of simple evidence-based interventions shown to prevent or treat the most important causes of newborn morbidity and mortality delivered at birth or in the early newborn period.^8^ EENC interventions include immediate and thorough drying; immediate and sustained skin-to-skin contact (SSC) for at least 90 min after delivery; delayed cord clamping; bag and mask resuscitation for non-breathing babies; timely initiation of and exclusive breastfeeding; appropriately timed hand-hygiene practices; management of newborn sepsis and limiting unnecessary caesarean sections, episiotomies, routine suctioning and admissions to neonatal care units.^9^ EENC also includes kangaroo mother care (KMC) and use of corticosteroids and magnesium sulfate for mothers in early labour to reduce respiratory and neurological complications.^10^

Implementation of EENC involved coaching health facility staff on appropriate childbirth and immediate newborn care practices using adult learning methodologies, focusing on practice in delivery rooms.^11 12^ Staff subsequently conducted periodic self-monitoring to identify and manage practice and systems gaps.^13^ In hospitals, multidisciplinary teams used a quality improvement approach including root-cause analysis to identify and address contextual factors that influence practice with available staff and resources. Trained and accredited hospital facilitators coached staff at their own and other facilities to scale-up the approach. Periodic external evaluations of a sample of national or subnational health facilities were conducted to assess progress and develop hospital and national action plans to improve quality.^14^ Initially introduced for term babies born vaginally, EENC was progressively expanded to include stable preterm/low birth weight (PTLBW) and caesarean births; KMC was introduced in 2017. Implementation was done with existing staff and facility systems using government funding, with periodic support by development partners for coaching and data collection.

By 2021–2022, estimated neonatal mortality declined to eight deaths per 1000 live births, a 70% reduction from 2010.^15^ In comparison, global neonatal mortality is estimated to have declined by 21.3% during the same period (from 22.5 to 17.7/1000 live births).^16^ While programme implementation of the EENC approach has been shown to improve newborn care practices and hospital outcomes, reduction of neonatal mortality at a national scale has not yet been demonstrated. This study aimed to determine the contribution of improved quality of early newborn care in Cambodia to the observed neonatal mortality decline.

## Methods

We adapted the evaluation framework for tracking MDG progress towards MDG goals to review the scale and effectiveness of EENC implementation to impact mortality.^17^ Implementation of EENC was assumed to have begun in 2012, the year of EENC clinical coaching scale-up in Cambodia. Standard indicators in the EENC monitoring and evaluation framework for tracking introduction and scale-up were used.^18^

Data on neonatal (0–28 days), postneonatal (28 days to 11 months), child (1–4 years) and maternal mortality between 2000 and 2022 were from Cambodia Demographic and Health Surveys (CDHS), conducted in 2000, 2010 and 2022 using standard methods and definitions.^15,19 20^ Average annual rates of mortality reduction were calculated for 2000–2010 and 2021–2022. Mortality data were disaggregated by economic quintile, residence, education and birth interval.

Population-based coverage of early newborn care interventions for 2000, 2010 and 2022 was estimated from the CDHS, including antenatal care (ANC), skilled and facility-based delivery, SSC between mother and baby (only for 2022), early breastfeeding (within 1 hour of birth) and exclusive breastfeeding, pre-lacteal and bottle feeding within 1 month of birth.

EENC programme implementation data were taken from submissions validated by the Regional EENC Independent Review Group.^21–23^ Routine programme data included EENC policies, strategies and plans; the proportion of facilities and staff receiving EENC coaching; and facility practice data from EENC facility assessments conducted 2015–2022 using standard WHO methods and tools and uploaded to the Regional WHO country database.^24^ Baseline observations done pre-coaching were used to estimate practices in 2011.^25^ CDHS measured breastfeeding within 60 min after birth, while facility assessments measured breastfeeding within 15–90 min, the EENC standard.

Facility assessments included randomly sampled national, provincial and district hospitals; and first-level facilities with at least 50 births per year that had introduced EENC. Standard checklists were used for interviewing mothers, observing births, reviewing maternal-newborn charts, hospital policies and environments and validating records.^14^ Births on the day of the visit were observed in sampled facilities using a standard checklist of 24 sequential practice steps required to deliver maternal and newborn interventions around childbirth.^11 14^ The standard facility assessment sampling approach was not used in 2015, with practice data collected from only two early implementation hospitals. 95% CIs around practice estimates were calculated using the Wilson score interval method to account for small sample proportions.^26^

We modelled newborn lives saved between 2010 and 2022 by EENC using the Lives Saved Tool (LiST) (Spectrum, V.6.36).^27^ Facility EENC practice assessments from 2015, 2017, 2019 and 2022 were used to estimate coverage of interventions delivered around birth. These were calculated as the product of the proportion of mothers and newborns receiving the intervention or clinical tasks required to deliver the intervention from facility assessment data and estimated proportion of facilities implementing EENC.^28^ Indicators included (1) care during labour and childbirth (clean birth environment, antenatal corticosteroids for preterm labour); (2) immediate newborn care (immediate drying, stimulation, thermal protection, ie, SCC and non-separation, delayed cord clamping, clean cord care, neonatal resuscitation); (3) early and exclusive breastfeeding (breastfeeding initiation at 15–90 min and exclusive breastfeeding at facility discharge); and (4) care of the small and ill neonate (management of newborn sepsis, care of preterm babies including KMC) (online supplemental tables 1-3).

Since limited routine assessment data were available for the management of newborn asphyxia, we applied management scores collected at supervisory follow-up visits conducted after EENC coaching at health facilities in 2012 using case simulations to estimate practice coverage.^29^ Similarly, no data were routinely collected on management of newborn sepsis at implementing health facilities; we used standard LiST assumptions for coverage of newborn sepsis management.^27,30^For all other assumptions of health status and for pre-conception, antenatal and pregnancy care interventions not included in the package of EENC interventions, standard LiST coverage assumptions were used. A sensitivity analysis of estimated deaths averted was conducted using the upper and lower 95% CI estimates around mean intervention coverage scores.

## Results

### Mortality trends

Neonatal mortality declined from 27/1000 live births in 2010 to 8/1000 live births in 2021–2022, a reduction of 70.4% across 12 years. The annual rate of reduction accelerated from 1.2% for 2000–2010 to 1.6% for 2011–2022, highest among rural, no education and low birth interval sub-groups. Postneonatal, child and maternal mortality all showed slowed rates of reduction between 2011 and 2022 (table 1).

**Table 1:** Neonatal, postneonatal, child and maternal mortality and rate of mortality reduction, stratified for socioeconomic and maternal characteristics, Cambodia demographic and health surveys, 2000, 2010 and 2022

Socioeconomic indicators and maternal characteristics	Mortality rates, n/1000 live births			Annual rate of reduction of mortality, %	
	2000	2010	2022	2000–2010	2011–2022
Neonatal mortality	39	27	8	1.2	1.6
Economic quintile					
Highest	–	16	5	–	0.9
Lowest	–	39	12	–	2.3
Residence					
Urban	27	11	6	1.6	0.4
Rural	41	35	10	0.6	2.1
Education					
Secondary+	27	20	5	0.7	1.3
No education	42	35	12	0.7	1.9
Birth interval (years)					
>4	23	22	9	0.1	1.1
<2	63	58	14	0.5	3.7
Postneonatal mortality	54	18	4	3.6	1.2
Economic quintile					
Highest	–	6	1	–	0.4
Lowest	…	38	10	…	2.3
Residence					
Urban	45	11	2	3.4	0.8
Rural	55	29	5	2.6	2.0
Education					
Secondary+	34	11	4	2.3	0.6
No education	60	37	16	2.3	1.8
Birth interval (years)					
>4	37	18	4	1.9	1.2
<2	71	55	21	1.6	2.8
Child mortality (1–4 years)	32	9	4	2.3	0.4
Economic quintile					
Highest	–	8	1	–	0.6
Lowest	–	15	5	–	0.8
Residence					
Urban	22	7	3	1.5	0.3
Rural	34	12	5	2.2	0.6
Education					
Secondary+	17	4	3	1.3	0.08
No education	37	16	7	2.1	0.8
Birth interval (years)					
>4	23	12	3	1.1	0.8
<2	45	19	8	2.6	0.9
Maternal mortality, n/100 000 live births	437	206	154	23.1	4.3

## Intervention coverage

Skilled birth attendance and facility births showed significant increases in coverage from 31.8% and 9.9% in 2000, to 98.7% and 97.8% respectively in 2022. The average rate of increase of both skilled and facility births for women without education rose significantly in 2011–2022 (table 2).

**Table 2:** Population coverage of selected maternal and newborn care interventions and rate of coverage change, stratified for socioeconomic characteristics, Cambodia Demographic and Health Surveys 2000, 2010 and 2022

Health service coverage	Coverage, %			Annual rate of increase	
	2000*	2010*	2022	2000–2010	2011–2022
Maternal					
Antenatal care 4+					
Mean	8.9	59.4	86.1	5.1	2.2
Highest	–	–	95.0	–	–
Lowest	–	–	74.2	–	–
Urban	–	80.3	91.3	–	0.9
Rural	–	55.3	82.8	–	2.3
Secondary+	–	–	90.0	–	–
No education	–	–	71.1	–	–
Skilled delivery					
Mean	31.8	71.0	98.7	3.9	2.3
Highest	–	96.7	100.0	–	0.3
Lowest	–	48.7	95.2	–	3.9
Urban	57.2	94.7	99.8	3.8	0.4
Rural	28.0	66.6	98.0	3.9	2.6
Secondary+	65.8	90.5	99.8	2.5	0.8
No education	19.3	46.9	93.3	2.8	3.9
Facility delivery					
Mean	9.9	53.8	97.8	4.4	3.7
Highest	–	87.5	99.6	–	1.0
Lowest	–	34.5	93.5	–	4.9
Urban	33.9	85.8	99.2	5.2	1.1
Rural	6.3	47.8	97.0	4.2	4.1
Secondary+	32.0	74.9	99.2	4.3	2.0
No education	3.3	33.9	91.8	3.1	4.8
C-section					
Mean	0.8	3.0	17.9	0.2	1.2
Highest	–	9.6	34.3	–	2.1
Lowest	–	1.1	8.6	–	0.6
Urban	3.2	8.2	23.9	0.5	1.3
Rural	0.5	2.0	14.1	0.2	1.0
Secondary+	3.4	6.3	21.0	0.3	1.2
No education	0.3	1.2	8.6	0.1	0.6
Neonatal					
Skin-to-skin contact					
Mean	0	0	76.8	0.0	6.4
Highest	0	0	66.4	0.0	5.5
Lowest	0	0	80.6	0.0	6.7
Urban	0	0	72.8	0.0	6.1
Rural	0	0	79.2	0.0	6.6
Secondary+	0	0	76.5	0.0	6.4
No education	0	0	75.6	0.0	6.3
Public	0	0	82.5	0.0	6.9
Private†	0	0	59.0	0.0	4.9
Early initiation of breastfeeding					
Mean	11.0	65.8	54.0	5.5	−1.0
Highest	–	62.0	34.3	–	−2.3
Lowest	–	65.4	71.0	–	0.5
Urban	13.2	64.4	49.5	5.1	−1.2
Rural	10.7	66.1	56.8	5.5	−0.8
Secondary+	14.7	65.0	52.7	5.0	−1.0
No education	12.0	63.8	53.7	5.2	−2.7
Exclusive breastfeeding<2 months	17.9	89.1	59.7	7.1	−2.5
Bottle feeding<2 months	9.9	6.5	32.8	−0.3	2.2
Postnatal care<2 days					
Mean	31.0	70.4	84.5	3.9	1.2
Highest		92.1	88.9	9.2	−0.3
Lowest		54.4	76.0	5.4	1.8
Urban	26.1	89.4	87.8	6.3	−0.1
Rural	31.8	66.8	82.4	3.5	1.3
Secondary+	28.9	85.3	86.4	5.6	0.1
No education	29.5	51.6	77.2	2.2	2.1

* (–) disaggregated data by economic quintile, residence and education not available.

† 68.6% of private medical providers are doctors, whereas 78.5% of public sector providers are midwives.

ANC, Antenatal care; PNC, Postnatal care.

The proportion of newborns receiving any SSC was 76.8% in 2022 and was not performed before 2011. Rates of application of SSC were highest in the lowest wealth quintile, rural populations and those born in public facilities. Early breastfeeding (<60 min) coverage rose from 11% in 2000 to 54.0% in 2022; and rates of exclusive breastfeeding rates in the first 2 months from 17.9% to 59.7% across the same period. The average annual rate of increase in early and exclusive breastfeeding declined in 2011–2022 compared with the previous decade.

### EENC programme implementation

Eight of nine WHO scale-up readiness benchmarks were achieved by 2022, with incorporation of EENC practice standards into pre-service training curricula not yet complete (online supplemental figure 1). By 2022, EENC was introduced in 88% of facilities, including 40.0% of national hospitals, 90% of provincial and district referral hospitals and 87% of primary care facilities providing birth services. An estimated 76.7% staff were coached in EENC by 2022 across all facility levels (online supplemental table 4). Facility assessments across sampled facilities and annual provincial planning reviews identified root causes of practice gaps in six key domains; data were used to plan actions that could be taken by staff, supervisors and facility managers using available resources and those that required provincial or national action (online supplemental figure 2).

### Policies, strategies, plans and guidelines

Between 2006 and 2008, several initiatives to strengthen availability and access to skilled provider care were initiated, including an increased midwife salary scale and midwifery incentive scheme. From 2012, EENC became the national early newborn care policy and was integrated into the minimum package of activities; policies, protocols and guidelines; and routine operational planning processes (online supplemental figure 3). National guidelines for management of newborn sepsis were endorsed and staff training instituted in 2015.^31^

### Facility practice standards

The average birth practice score for breathing babies by direct observation was 72.9% in 2022, rising from 6% in 2011 (table 3). By 2022, interview data showed significant improvements in proportion of both term and PTLBW newborns receiving any SSC, initiating SSC within 1 min of birth, having SSC uninterrupted for at least 60 min and breastfeeding early and exclusively (table 4). Practice coverage was generally higher for term than PTLBW newborns. From 2017, an increasing proportion of babies born by caesarean section received SSC. Significant improvements in use of corticosteroids for mothers in preterm labour and KMC for babies less than 2000 g were noted by 2022 (table 4). Improvements in facility-based practice estimates tracked mortality declines across the 10-year implementation period (online supplemental figure 4).

**Table 3:** Early essential newborn care clinical practice scores, birth observations, facility-based implementation assessments, Cambodia, 2011–2022

Activity	Year				
	2011, % (n/10) (95% CI)	2015, % (n/32) (95% CI)	2017, % (n/11) (95% CI)	2019, % (n/26) (95% CI)	2022, % (n/23) (95% CI)
Pre-birth preparation					
1. Checked room temperature (>25°C); turned off fans	–	90.6 (29) (75.8 to 96.8)	63.6 (7)(35.4 to 84.8)	73.1 (19)(53.9 to 86.3)	73.9 (17) (53.5 to 87.5)
2. Washed hands (first time)	0.0 (0) (0.0 to 27.8)	100.0 (32) (89.3 to 100.0)	90.9 (10) (62.3 to 98.4)	76.9 (20) (57.9 to 89.0)	87.0 (20) (67.9 to 95.5)
3. Dry cloth placed on abdomen	–	100.0 (32) (89.3 to 100.0)	81.8 (9) (52.3 to 94.9)	57.7 (15) (38.9 to 74.5)	87.0 (20) (67.9 to 95.5)
4. Prepared the newborn resuscitation area	0.0 (0) (0.0 to 27.8)	100.0 (32) (89.3 to 100.0)	90.9 (10) (62.3 to 98.4)	50.0 (13) (32.1 to 67.9)	78.3 (18) (58.1 to 90.3)
5. Checked if bag and mask are functional	0.0 (0) (0.0 to 27.8)	81.2 (26) (64.7 to 91.1)	54.5 (6) (28.0 to 78.7)	50.0 (13) (32.1 to 67.9)	65.2 (15) (44.9 to 81.2)
6. Suction bulb on delivery table, checked for functionality	0.0 (0) (0.0 to 27.8)	87.5 (28) (71.9 to 95.0)	63.6 (7) (35.4 to 84.8)	61.5 (16) (42.5 to 77.6)	69.6 (16) (49.1 to 84.4)
7. Washed hands (second time)	0.0 (0) (0.0 to 27.8)	81.2 (26) (64.7 to 91.1)	54.5 (6) (28.0 to 78.7)	73.1 (19) (53.9 to 86.3)	60.9 (14) (40.8 to 77.8)
8. Wore two pairs of clean gloves (if only one health worker)	0.0 (0) (0.0 to 27.8)	93.8 (30) (79.9 to 98.3)	100.0 (11)(74.1 to 100.0)	88.5 (23) (71.0 to 96.0)	91.3 (21) (73.2 to 97.6)
9. Arranged forceps, cord clamp/ties in easy to use order	0.0 (0) (0.0 to 27.8)	90.6 (29) (75.8 to 96.8)	90.9 (10) (62.3 to 98.4)	73.1 (19) (53.9 to 86.3)	78.3 (18) (58.1 to 90.3)
Immediate postpartum					
10. Called out time of birth (hours, minutes, seconds)	0.0 (0) (0.0 to 30.8)	81.2 (26) (64.7 to 91.2)	54.5 (6) (25.6 to 81.2)	76.9 (20) (56.4 to 89.8)	87.0 (20) (67.0 to 95.5)
11. Drying started within 5 s after birth *Answer<5 s (Y), 5–10 s (P), >10 s (N)	0.0 (0) (0.0 to 27.8)	56.2 (18) (39.3 to 71.8)	45.5 (5) (21.3 to 72.0)	57.7 (15) (38.9 to 74.5)	69.6 (16) (49.1 to 84.4)
12. Dried the baby thoroughly (wipe eyes, face, head, front, back, arms and legs)	0.0 (0) (0.0 to 27.8)	65.6 (21) (48.3 to 79.6)	72.7 (8) (43.4 to 90.3)	100.0 (26) (87.1 to 100.0)	95.7 (22) (79.0 to 99.2)
13. Assessed the baby’s breathing, colour, reflex and tone while drying	–	96.9 (31) (84.3 to 99.4)	100.0 (11) (74.1 to 100.0)	69.2 (18) (50.0 to 83.5)	56.5 (13) (36.8 to 74.4)
14. Removed the wet cloth	0.0 (0) (0.0 to 27.8)	93.8 (30) (79.9 to 98.3)	63.6 (7) (35.4 to 84.8)	84.6 (22) (66.5 to 93.9)	87.0 (20) (67.9 to 95.5)
15. Placed baby in direct skin-to-skin contact	0.0 (0) (0.0 to 27.8)	96.9 (31) (84.3 to 99.4)	90.9 (10) (62.3 to 98.4)	96.2 (25) (81.1 to 99.3)	91.3 (21) (73.2 to 97.6)
16. Covered baby’s body with cloth and head with a hat	0.0 (0) (0.0 to 27.8)	84.4 (27) (68.2 to 93.1)	100.0 (11) (74.1 to 100.0)	84.6 (22) (66.5 to 93.9)	73.9 (17) (53.5 to 87.5)
17. Checked for a second baby	–	100.0 (32) (89.3 to 100.0)	90.9 (10) (62.3 to 98.4)	84.6 (22) (66.5 to 93.9)	91.3 (21) (73.2 to 97.6)
18. Oxytocin given to mother within 1 min	–	100.0 (32) (89.3 to 100.0)	90.9 (10) (62.3 to 98.4)	92.3 (24) (75.9 to 97.9)	95.7 (22) (79.0 to 99.2)
19. First pair of gloves removed	0.0 (0) (0.0 to 27.8)	87.5 (28) (71.9 to 95.0)	72.7 (8) (43.4 to 90.3)	88.5 (23) (71.0 to 96.0)	82.6 (19) (62.9 to 93.0)
20. Cord pulsations checked before clamping, clamped after cord pulsations stopped	0.0 (0) (0.0 to 27.8)	96.9 (31) (84.3 to 99.4)	100.0 (11) (74.1 to 100.0)	61.5 (16) (42.5 to 77.6)	73.9 (17) (53.5 to 87.5)
21. Clamp/tie placed at 2 cm, forceps at 5 cm from newborn’s abdomen and cord cut between the clamps/ties	10.0 (1) (1.8to 40.4)	96.9 (31) (84.3 to 99.4)	81.8 (9) (52.3 to 94.9)	92.3 (24) (75.9 to 97.9)	100.0 (23) (85.7 to 100.0)
22. Delivered placenta	–	78.1 (25) (61.2 to 89.0)	63.6 (7) (35.4 to 84.8)	84.6 (22) (66.5 to 93.9)	95.7 (22) (79.0 to 99.2)
23. Counselled mother on feeding cues *Answer>2 cues (Y), 1–2 cues (P)	0.0 (0) (0.0 to 27.8)	78.1 (25) (61.2 to 89.0)	90.9 (10) (62.3 to 98.4)	61.5 (16) (42.5 to 77.6)	69.6 (16) (49.1 to 84.4)
24. Monitored the vital signs of mother and newborn every 15 min in first hour	–	100.0 (32) (89.3 to 100.0)	81.8 (9) (52.3 to 94.9)	76.9 (20) (57.9 to 89.0)	56.5 (13) (36.8 to 74.4)
Average score (%, n/N): (maximum 48)*	6.2 (2) (1.7 to 20.1)	87.5 (42) (75.3 to 94.1)	85.4 (41) (72.8 to 92.8)	70.8 (34) (56.8 to 81.8)	72.9 (35) (59.0 to 83.4)

* 24 standard practices were scored as yes (two points), partial (one point) and no (zero points), with the maximum possible score for all practices 48. Pre-coaching observations in 2011 used a reduced 16-point practice checklist with a total possible score of 32.

**Table 4:** Birth and early newborn care practices, maternal exit interviews and chart reviews, facility-based implementation assessments, Cambodia, 2015–2022

Early essential newborn care indicator	2015, % (n) (95% CI)	2017, % (n) (95% CI)	2019, % (n) (95% CI)	2022, % (n) (95% CI)
Birth practices—term babies				
Partograph completed (term)	100.0 (13) (77.2 to 100.0)	77.6 (118) (70.4 to 83.5)	56.5 (135) (50.1 to 62.6)	66.8 (169) (60.8 to 72.3)
Food and fluids on demand	–	97.7 (130) (93.6 to 99.2)	92.4 (195) (88.0 to 95.3)	93.1 (201) (88.9 to 95.7)
Companion of choice	–	89.5 (119) (83.1 to 93.6)	93.8 (198) (89.7 to 96.4)	88.4 (191) (83.5 to 92.0)
Position of choice	100.0 (18) (82.4 to 100.0)	95.5 (127) (90.5 to 97.9)	93.8 (198) (89.7 to 96.4)	93.1 (201) (88.9 to 95.7)
Fundal pressure	12.5 (2) (3.5 to 36.0)	3.8 (5) (1.6 to 8.5)	16.6 (35) (12.2 to 22.2)	5.1 (11) (2.9 to 8.9)
Enema	–	1.3 (2) (0.4 to 4.7)	7.9 (19) (5.1 to 12.1)	2.4 (6) (1.1 to 5.1)
SSC, any				
Total	83.3 (15) (60.8 to 94.2)	92.1 (151) (86.9 to 95.3)	90.4 (235) (86.2 to 93.4)	92.1 (267) (88.4 to 94.7)
Term	–	94.1 (143) (89.1 to 96.9)	91.2 (218) (86.9 to 94.2)	94.1 (238) (90.4 to 96.4)
Preterm/LBW*	–	66.7 (8) (39.1 to 86.2)	81.0 (17) (60.0 to 92.3)	78.4 (29) (62.8 to 88.6)
SSC<1 min of birth				
Total	50.0 (9) (29.0 to 71.0)	74.4 (122) (67.2 to 80.5)	83.1 (216) (78.0 to 87.1)	77.6 (225) (72.4 to 82.0)
Term	50.0 (9) (29.0 to 71.0)	75.0 (114) (67.6 to 81.2)	84.9 (203) (79.9 to 88.9)	79.1 (200) (73.6 to 83.6)
Preterm/LBW	–	66.7 (8) (39.1 to 86.2)	61.9 (13) (40.9 to 79.2)	67.6 (25) (51.5 to 80.4)
Duration of SSC				
Term				
None	16.7 (3) (5.8 to 39.2)	5.9 (9) (3.1 to 10.9)	8.8 (21) (5.8 to 13.1)	5.9 (15) (3.6 to 9.6)
0–59 min	5.6 (10) (33.7 to 75.4)	71.7 (109) (64.1 to 78.3)	68.2 (163) (62.0 to 73.8)	77.9 (197) (72.4 to 82.5) (72.4 to 82.5)
60–89 min	27.8 (5) (12.5 to 50.9)	12.5 (19) (8.2 to 18.7)	13.0 (31) (9.3 to 17.8)	6.7 (17) (4.2 to 10.5)
≥90 min	–	9.9 (15) (6.1 to 15.6)	10.0 (24) (6.8 to 14.5)	9.5 (24) (6.5 to 13.7)
Preterm/LBW				
None	–	33.3 (4) (13.6 to 62.4)	19.0 (4) (7.7 to 40.0)	21.6 (8) (11.5 to 37.2)
0–59 min	50.0 (6) (25.4 to 74.6)	71.4 (15) (49.8 to 86.8)	59.5 (22) (43.3 to 74.1)	50.0 (6) (25.4 to 74.6)
60–89 min	–	16.7 (2) (4.7 to 44.8)	0.0 (0) (0.0 to 15.5)	13.5 (5) (5.9 to 27.8)
≥90 min	–	0.0 (0) (0.0 to 24.7)	9.5 (2) (2.6 to 28.9)	5.4 (2) (1.5 to 17.7)
SSC, until completed breastfeed				
Total	–	47.6 (78) (40.1 to 55.2)	56.2 (146) (50.1 to 62.1)	32.4 (94) (27.3 to 38.0)
Term	–	49.3 (75) (41.5 to 57.2)	59.0 (141) (52.7 to 65.0)	34.8 (88) (29.2 to 40.8)
Preterm/LBW	–	25.0 (3) (8.9 to 53.2)	23.8 (5) (10.6 to 45.1)	16.2 (6) (7.7 to 31.1)
SCC with caesarean section				
Total	–	75.0 (15) (53.1 to 88.8)	43.3 (13) (27.4 to 60.8)	67.4 (29) (52.5 to 79.5)
Term	–	78.9 (15) (56.7 to 91.5)	46.4 (13) (29.5 to 64.2)	70.3 (26) (54.2 to 82.5)
Preterm/LBW	–	0.0 (0) (0.0 to 79.3)	0.0 (0) (0.0 to 65.8)	50.0 (3) (18.8 to 81.2)
Breastfeeding, any				
Total	88.2 (15) (65.7 to 96.7)	93.3 (153) (88.4 to 96.2)	89.6 (233) (85.3 to 92.8)	92.8 (269) (89.2 to 95.2)
Term	–	95.4 (145) (90.8 to 97.8)	92.1 (220) (87.9 to 94.9)	92.9 (235) (89.0 to 95.5)
Preterm/LBW	–	66.7 (8) (39.1 to 86.2)	61.9 (13) (40.9 to 79.2)	91.9 (34) (78.7 to 97.2)
Breastfeed, timely initiation 15–90 min†				
Total	26.7 (4) (10.9 to 52.0)	59.1 (97) (51.5 to 66.4)	49.2 (128) (43.2 to 55.3)	53.4 (155) (47.7 to 59.1)
Term	26.7 (4) (10.9 to 52.0)	61.2 (93) (53.3 to 68.6)	51.5 (123) (45.2 to 57.7)	53.4 (135) (47.2 to 59.4)
Preterm/LBW	–	33.3 (4) (13.8 to 60.9)	23.8 (5) (10.6 to 45.1)	54.1 (20) (38.4 to 69.0)
Exclusive breastfeeding at discharge				
Total	77.8 (14) (54.8 to 91.0)	86.0 (141) (79.8 to 90.5)	81.5 (212) (76.4 to 85.8)	91.7 (266) (88.0 to 94.4)
Term	–	87.5 (133) (81.3 to 91.8)	82.4 (197) (77.1 to 86.7)	92.5 (234) (88.6 to 95.1)
Preterm/LBW	–	66.7 (8) (39.1 to 86.2)	71.4 (15) (50.0 to 86.2)	86.5 (32) (72.0 to 94.1)
Fed from a bottle				
Total	11.1 (2) (3.1 to 32.8)	13.4 (22) (9.0 to 19.5)	18.1 (47) (13.9 to 23.2)	13.4 (39) (10.0 to 17.9)
Term	–	12.5 (19) (8.2 to 18.7)	17.2 (41) (12.9 to 22.4)	11.5 (29) (8.1 to 16.0)
Preterm/LBW	–	25.0 (3) (8.9 to 53.2)	28.6 (6) (13.8 to 50.0)	27.0 (10) (15.4 to 43.0)
Early bathing	–	11.2 (17) (7.1 to 17.2)	16.7 (40) (12.5 to 22.0)	8.7 (22) (5.8 to 12.8)
24–34-week GA received corticosteroids				
First dose within 1 hour of arrival	–	28.6 (2) (8.2 to 64.1)	85.7 (12) (60.1 to 96.0)	75.0 (9) (46.8 to 91.1)
Full course	–	57.1 (8) (32.6 to 78.6)	75.0 (9) (46.8 to 91.1)	–
<2000 g received KMC				
Any	–	0.0 (0) (0.0 to 35.4)	61.1 (11) (38.6 to 79.7)	40.0 (4) (16.8 to 68.7)
Continuous	–	0.0 (0) (0.0 to 35.4)	5.6 (1) (1.0 to 25.8)	0.0 (0) (0.0 to 27.8)

* GA of <37 completed weeks and/or <2500 g birth weight.

† Timely initiation in 2015 was defined as initiation between 15 and 60 min.

GA, gestational age; KMC, kangaroo mother care; LBW, low birth weight; SSC, skin-to-skin contact.

### LiST modelling

LiST modelling using mean facility coverage estimates found that between 2010 and 2022, EENC averted 21 704 newborn deaths, representing 84.5% of 25 670 newborn deaths averted by the full package of pregnancy, childbirth and post-birth preventive and curative interventions during this period (table 5). The model projected a newborn mortality rate in 2022 of 12.7/1000, a decline of 14.3/1000 live births over 12 years. This represented 75.3% of the observed mortality decline calculated from CDHS data (19/1000 live births across 12 years). The model did not account for 24.7% of the observed mortality decline. Sensitivity analysis using upper and lower 95% CI coverage estimates found that EENC interventions averted 81.5–87.1% of all deaths averted by the complete package of interventions over 12 years. Using upper and lower bound values, newborn mortality at the end of the intervention period was estimated to be between 13.8/1000 (69.5% of the observed mortality decline) to 11.7/1000 live births (80.5% of the observed mortality decline) (table 5).

**Table 5:** Estimated practice coverage and newborn deaths averted by EENC interventions in the birth and early newborn period using the LiST, Cambodia, 2011–2022

EENC intervention or intervention package	Estimated practice coverage, % (95% CI)					Estimated deaths averted 2010–2022 Mean (lower–upper)
	2011	2015	2017	2019	2022	
Clean birth environment	0 (0 to 27.8)	83.5 (70.9 to 87.5)	68.3 (44.1 to 80.0)	59.7 (43.0 to 71.9)	67.5 (50.2 to 78.7)	688 (550–851)
Immediate drying and stimulation	0 (0 to 27.8)	66.3 (52.2 to 76.8)	64.7 (40.5 to 78.2)	67.3 (60.5 to 76.5)	65.0 (48.5 to 75.2)	2401 (1866–2902)
Thermal protection	0 (0 to 27.8)	75.8 (55.6 to 85.7)	82.0 (77.6 to 84.8)	81.8 76.7to83.1)	81.0 (77.7 to 83.3)	3721 (4084)
Delayed cord clamping	5 (1.8 to 34.1)	88.2 (76.8 to 91.0)	80.9 (56.6 to 85.0)	68.4 (53.0 to 76.9)	76.6 (61.2 to 82.3)	4450 (3643–5025)
Clean cord care	0 (0 to 27.8)	82.5 (69.4 to 87.0)	72.8 (49.0 to 85.0)	71.2 (54.7 to 80.1)	70.4 (53.6 to 79.6)	1085 (870–1293)
Neonatal resuscitation	5 (1.8 to 34.1)	82.3 (77.4 to 85.5)	80.5 (75.7 to 83.8)	80.5 (75.7 to 83.8)	79.6 (74.8 to 82.7)	5188 (5155–5836)
Early initiation of breastfeeding 15–90 min	65.7 (64.0 to 67.4)	24.3 (10.0 to 47.3)	52.6 (46.3 to 59.1)	43.8 (38.3 to 82.6)	47.0 (42.2 to 83.8)	682 (−101 to 1270)
Exclusive breastfeeding at facility discharge	73.4 (70.0 to 76.6)	70.8 (52.1 to 82.8)	76.5 (71.2 to 80.6)	72.5 (67.6 to 76.4	80.7 (77.4 to 82.8)	
Corticosteroids for premature babies (24–34 weeks)	0	0	25.5 (7.1 to 57.1)	76.3 (53.4 to 85.4)	66.0 (41.4 to 80.2)	1086 (732–1853)
Case management of neonatal sepsis	26.3 (19.7 to 32.9)	45.7 (34.3 to 57.1)	47.9 (35.9 to 59.9)	50.1 (37.6 to 62.6)	52.3 (39.2 to 65.4)	1597 (1246–1948)
KMC for premature babies <2000 g	0	0	0 (0 to 38.7)	54.4 (34.7 to 69.2)	35.2 (15.0 to 61.6)	806 (397–1388)
Total						21 704* (17 694–26 450)

* Total deaths averted by the full package of pregnancy, childbirth and post-birth preventive and curative interventions included in the LiST model: n=25 670 (mean coverage), n=21 708 (lower bound coverage estimates), n=30 375 (upper bound coverage estimates).

EENC, early essential newborn care; KMC, kangaroo mother care; LiST, Lives Saved Tool.

## Discussion

### Association between mortality declines and EENC

EENC interventions averted an estimated 21 704 Cambodian newborn deaths during 2010–2022 and were responsible for 75.3% of the observed neonatal mortality reduction nationally. The average annual rate of neonatal mortality decline accelerated by 33% during this period compared to the 10-year period preceding EENC, with greatest declines among high-risk groups (poorest, rural, uneducated and with low birth interval). In contrast, rates of postneonatal, child and maternal mortality decelerated, suggesting that other unmeasured factors operating either within or outside the health system were less likely to be contributing to observed neonatal mortality decline.

A prerequisite for EENC mortality impact is improved access to and availability of services. EENC was scaled-up rapidly from 2012, with EENC facility and health worker coverage rising to 88% and 77%, respectively, by 2022. Concurrently, skilled birth attendance rates rose to 98.7% in 2022, with average annual rates of increase highest in uneducated, low income and rural populations at highest risk. Facility-based birth observations showed significant improvements in quality of early newborn care practices during 2011–2022, including application of clean birth environments, immediate drying and stimulation, thermal protection (immediate and prolonged SSC), delayed cord clamping and clean cord care. Around half of term and PTLBW babies breastfed in the first 15–90 min at facilities in 2022; and 92.5% and 86.5%, respectively, exclusively breastfed on discharge. Practice coverage was highest for term newborns. However, practice improvements for PTLBW and caesarean births were also significant, with KMC coverage rising to 40% in 2022; previously these vulnerable newborns were historically separated for observation, given formula and admitted to neonatal care units (NCUs), putting them at higher risk of complications and death. Findings are consistent with those from other EENC implementing countries showing that EENC can be implemented at scale and improves health worker practices.^32–34^ Increasing durations of SSC up to 90 min have a strong dose–response relationship with early and exclusive breastfeeding.^35 36^ EENC has been demonstrated to increase the uptake of KMC.^37^ Improvements in skilled and facility birth rates were driven by financial support mechanisms that improved access.^38 39^

Plausibility that mortality reduction was linked to increased coverage of EENC interventions is supported by a substantial evidence base.^9^ Delivery of EENC across the region is associated with reductions in unnecessary NCU admissions, hypothermia, sepsis and other morbidity, including high-risk newborns born PTLBW or by caesarean section.^40–42^ The benefits of early and exclusive breastfeeding on newborn morbidity and mortality are well established.^43^ PTLBW babies managed with KMC have at least a 40% reduced risk of mortality.^44^ Mortality declines among subpopulations at highest risk in Cambodia (poor, rural and less educated) tracked improved access to birth care in the same groups, further strengthening the association with improved care quality.

### Limitations

Attribution of newborn lives saved is dependent on estimates of intervention efficacy and coverage used in the model.^45^ Estimates of facility practice coverage in Cambodia assumed that survey data were representative of birth practices across national, provincial and district hospitals and primary birth facilities. Birth facilities that saw fewer than 50 births per year were excluded from facility samples because low birth numbers compromised validity and reliability of data; however, these facilities represented a small fraction of facilities. Larger facility sample sizes would have increased validity and reliability of estimates, but were not feasible for routine programme use. More accurate coverage estimates could be obtained by linking facility practice data with place of delivery, but this linkage was not possible with available data and is recommended for future analyses. Small sample sizes for some variables resulted in relatively wide CIs; however modelling using upper and lower bound coverage estimates found that EENC interventions accounted for 69–82% of the observed mortality reduction over 12 years. Assumptions for coverage of resuscitation and management of neonatal sepsis for this analysis were based on best available data and standard estimates used by LiST.^27 30 46^ Better practice data are needed in these areas for future modelling. The LiST model may underestimate the impact of some EENC interventions not included in the standard model including prolonged SSC, non-separation to warmers, elimination of routine suction and non-touching policies and interventions in the second stage of labour.^47^The fraction of newborn mortality decline not measured by the LiST model is therefore likely to represent both unmeasured intervention impact and factors operating outside of the health system.^48^

Periodic cross-sectional facility practice assessments may not represent quality of services across time, which may change with case loads, case type, availability of staff or staff skills. Observation of staff birth practices are subject to the Hawthorne effect. Maternal exit interviews are subject to recall and response bias. To mitigate bias, facilities were not informed in advance of evaluation visits, observed births were sampled randomly where possible, and maternal interviews conducted on a systematic sample of newborns delivered in the previous 24 hours to reduce observation bias and obtain information on practices outside the delivery room. Validation of mothers’ recall of immediate newborn care practices has shown high levels of agreement between observed and reported measures.^33^ Further, the same tools and methods were used over time, reducing impact on trends. We assumed that facility-based coverage of early SSC, early breastfeeding and exclusive breastfeeding at discharge, used for estimating deaths averted, are better measures of potential contribution to mortality reduction because newborn deaths are concentrated in the first days of life. Standard programme tools did not allow measurement of additional factors that may influence SSC and breastfeeding, including education, income, maternal infant-feeding intention prior to birth, smoking, race and ethnicity and intrapartum analgesia and anaesthesia. These factors may warrant further study to enable better targeting of practice barriers.

### Lessons learned for wider use

Programmatic shifts required to institutionalise EENC in Cambodia have been implemented across nine countries in East Asia and the Pacific, suggesting that the approach is feasible in a range of different system contexts.^23 49^ The introduction of coaching using local facilitators was central to improving quality and ownership, because it allowed practical skills to be tailored to practice environments, organisation and workflow; and barriers to practice addressed by dialogue with practitioners. Findings are consistent with previous data suggesting that case-based learning and clinical simulations with frequent repetition are effective at changing clinical practices, particularly when conducted in settings similar to the workplace.^50^ Adult learning methods including experiential learning, question asking, immediate feedback and group dialogue to allow exchange of experience and problem solving have been demonstrated to improve acquisition of knowledge and skills.^51 52^ Over-collection and processing of unnecessary data and unsustainable complex processes were consciously avoided.^53^ Frontline staff were facilitated to identify actions they could take themselves to address common barriers. Multidisciplinary teams including senior leadership put organisational change at the centre of efforts to improve practice quality, in tandem with improved patient and provider education.^54^

The introduction of two yearly rapid national practice evaluations was central to institutionalisation. Assessments established for the first time indicators for tracking progress, allowed data to be compared between facility levels, engaged national and subnational staff collaboratively in data collection, analysis and interpretation and allowed evidence-based planning. Similarly, scale-up readiness benchmarks provided quantifiable measures for programme integration of EENC including mechanisms for technical and stakeholder coordination and consensus-based policy and guideline endorsement. EENC was primarily implemented through existing government systems with local resources; decentralised annual planning in Cambodia allowed provincial data and experience to inform priorities and resource allocations. However, small group coaching required a larger number of facilitators than centralised traditional training and increases the time required to reach all staff; this must be factored into planning for scale-up.

Several issues require continued attention to ensure sustainability. Although EENC standards and coaching methods have been incorporated into some pre-service curricula, the quality and extent remain uncertain. Pre-service curriculum reviews and updates are difficult because of the numerous pre-service educational institutions and the time and coordination required. Harmonising and periodically updating the content and methods used for pre-service education remains a priority for sustained practice change in many LMICs including Cambodia.^55^

Practice gaps remain for babies born PTLBW or by caesarean section and continuous KMC remains suboptimal. Ensuring uninterrupted SSC for at least 90 min is a challenge in birth environments. Skill decay is a potential problem over time due to staff turnover. It remains critical that local barriers to practice are identified and targeted through practice observations, feedback and root-cause analysis. Early and unnecessary mother-baby separations are often attributed to availability of space, staff time limitations, concerns about privacy, family fears and resistance by senior staff.^23 33^ Reasons postulated for why SSC and early newborn care practices are not performed at caesarean section births include fears that it will compromise the sterile field, concerns for the safety of mothers and babies, perceived additional work, physical organisation of the operating room and poor coordination and communication between anaesthesiology and obstetrics staff. These concerns often persist despite evidence that it is feasible, effective and well-accepted by mothers.^56^ Although high rates of exclusive breastfeeding at discharge are noted, maintaining exclusive breastfeeding practices after discharge is increasingly difficult in Cambodia.^57^ As in much of Asia, numerous risks to breastfeeding are emerging that require enhanced multi-sectoral effort, including more attention to adoption and active enforcement of the International Code of Marketing of Breast-milk Substitutes.^58^

## Conclusion

A new approach to EENC programme implementation in Cambodia led to sustained country-wide improvements in evidence-based practices around birth. Improved care quality was associated with 69.5–80.5% of the observed neonatal mortality decline in Cambodia between 2010 and 2022.

## Supplementary material

10.1136/bmjoq-2025-003920 online supplemental figure 1

10.1136/bmjoq-2025-003920 online supplemental figure 2

10.1136/bmjoq-2025-003920 online supplemental figure 3

10.1136/bmjoq-2025-003920 online supplemental figure 4

10.1136/bmjoq-2025-003920 online supplemental file 1

## Data Availability

Data are available upon reasonable request.
