# Supplementary material for: Contribution of improved quality of early essential newborn care to neonatal mortality decline in Cambodia, 2010–2022: a retrospective observational study
Source: BMJ Open Qual. 2026 Sep 1;15(3):e003920. doi: 10.1136/bmjoq-2025-003920 (PMC13536012; doi:10.1136/bmjoq-2025-003920)

Supplemental Figure 1: Status of EENC scale-up readiness benchmarks, Cambodia, 2010 - 2022

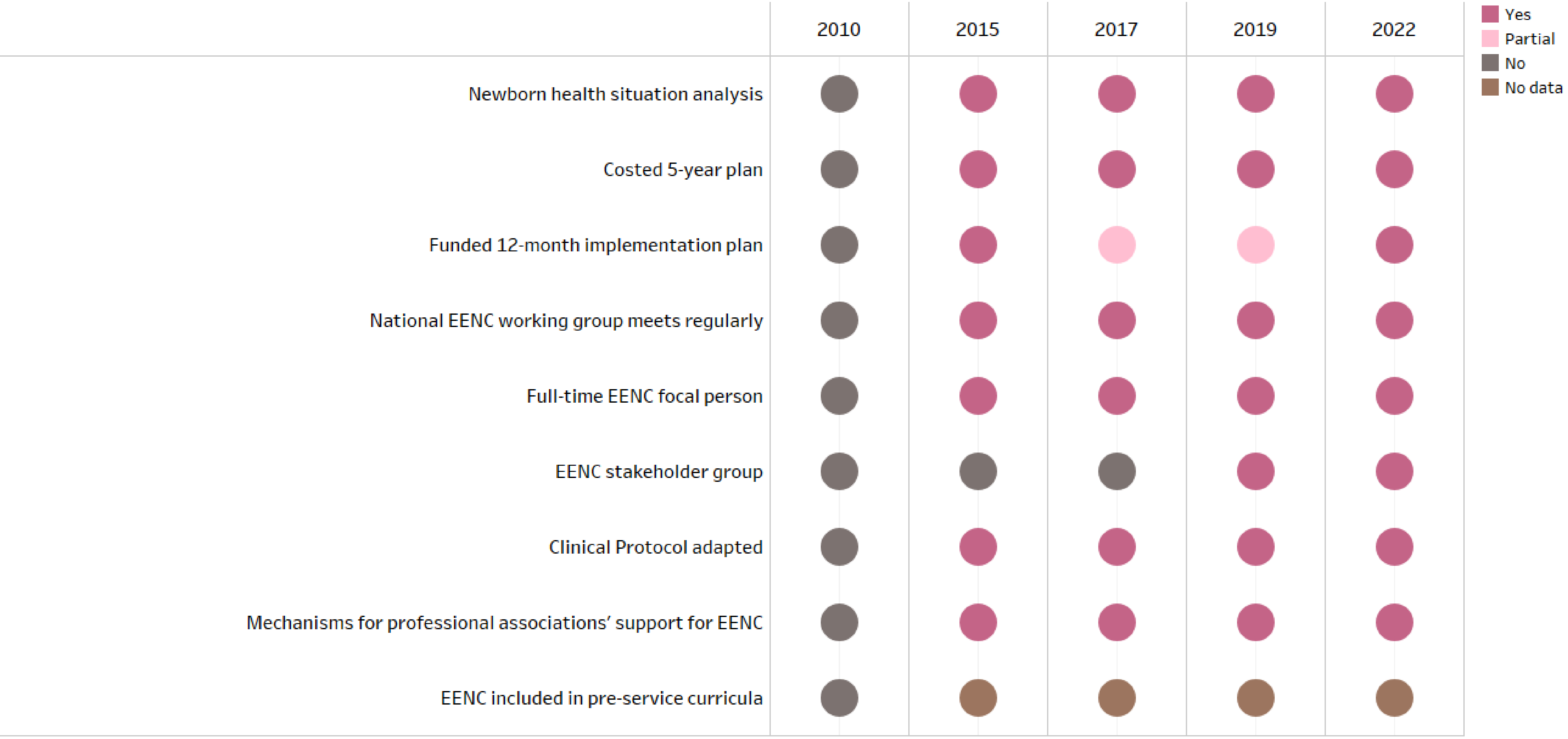

Supplement: Supplementary file 1 [file bmjoq-15-3-s001.pdf]
