# Supplementary material for: Contribution of improved quality of early essential newborn care to neonatal mortality decline in Cambodia, 2010–2022: a retrospective observational study
Source: BMJ Open Qual. 2026 Sep 1;15(3):e003920. doi: 10.1136/bmjoq-2025-003920 (PMC13536012; doi:10.1136/bmjoq-2025-003920)

Supplemental Figure 2: Root cause analysis for EENC practice gaps at implementing facilities, facility-based implementation reviews, Cambodia, 2011-2022

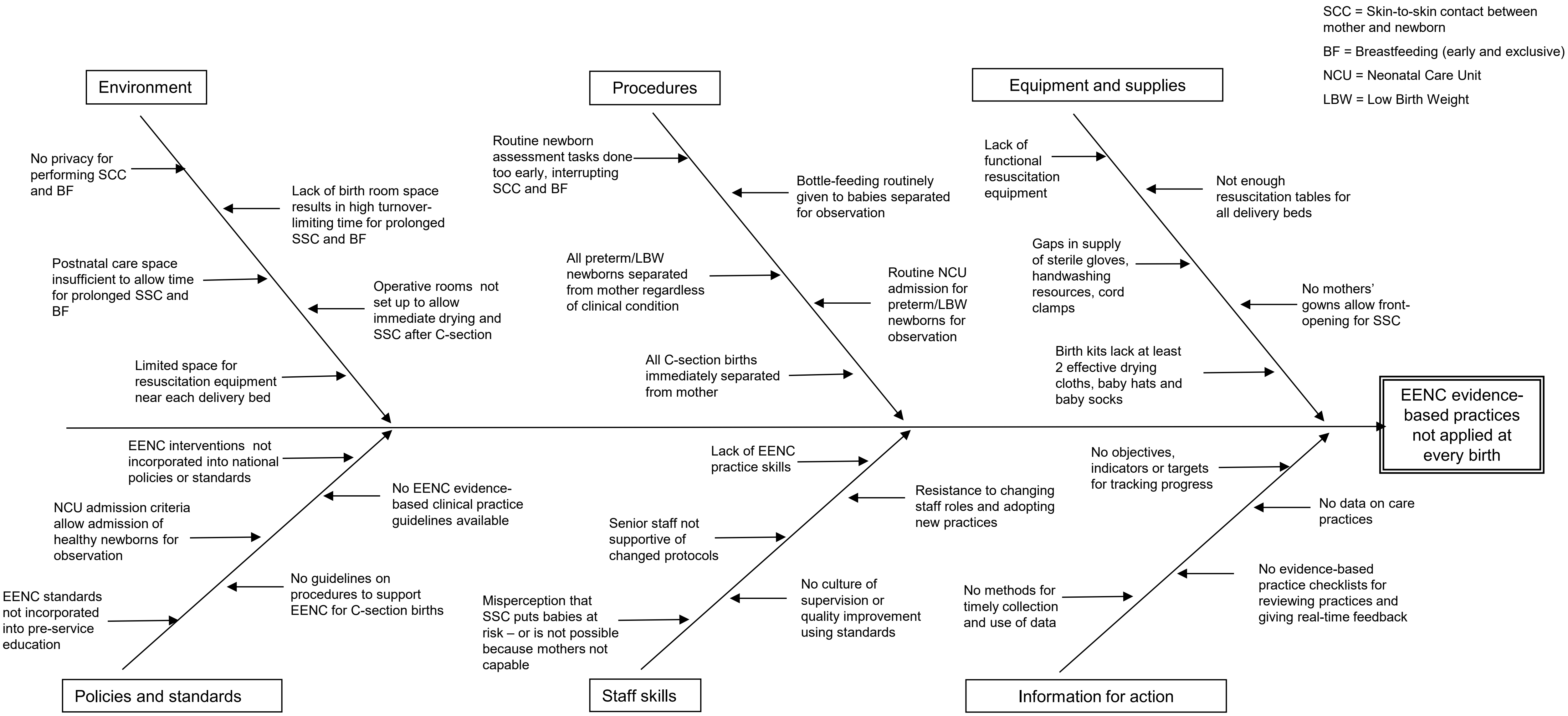

Supplement: Supplementary file 2 [file bmjoq-15-3-s002.pdf]
