# Supplementary material for: Contribution of improved quality of early essential newborn care to neonatal mortality decline in Cambodia, 2010–2022: a retrospective observational study
Source: BMJ Open Qual. 2026 Sep 1;15(3):e003920. doi: 10.1136/bmjoq-2025-003920 (PMC13536012; doi:10.1136/bmjoq-2025-003920)

Supplemental Figure 3: Policies, strategies, initiatives, incentive schemes and programmatic inputs and interventions, Cambodia, 2000-2022.

(a) Policy support for skilled delivery

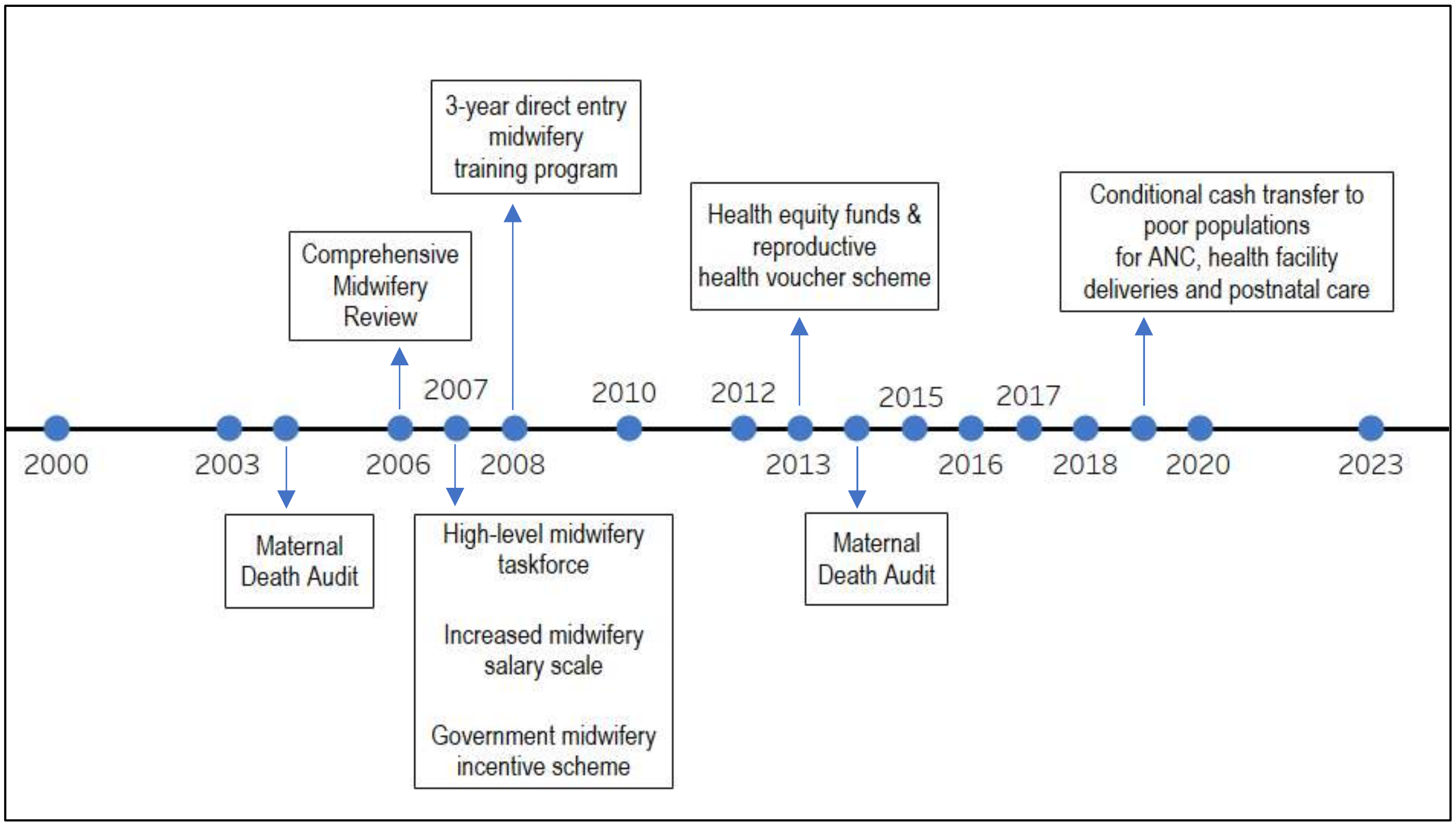

(b) Incorporation of EENC into national policies and strategies

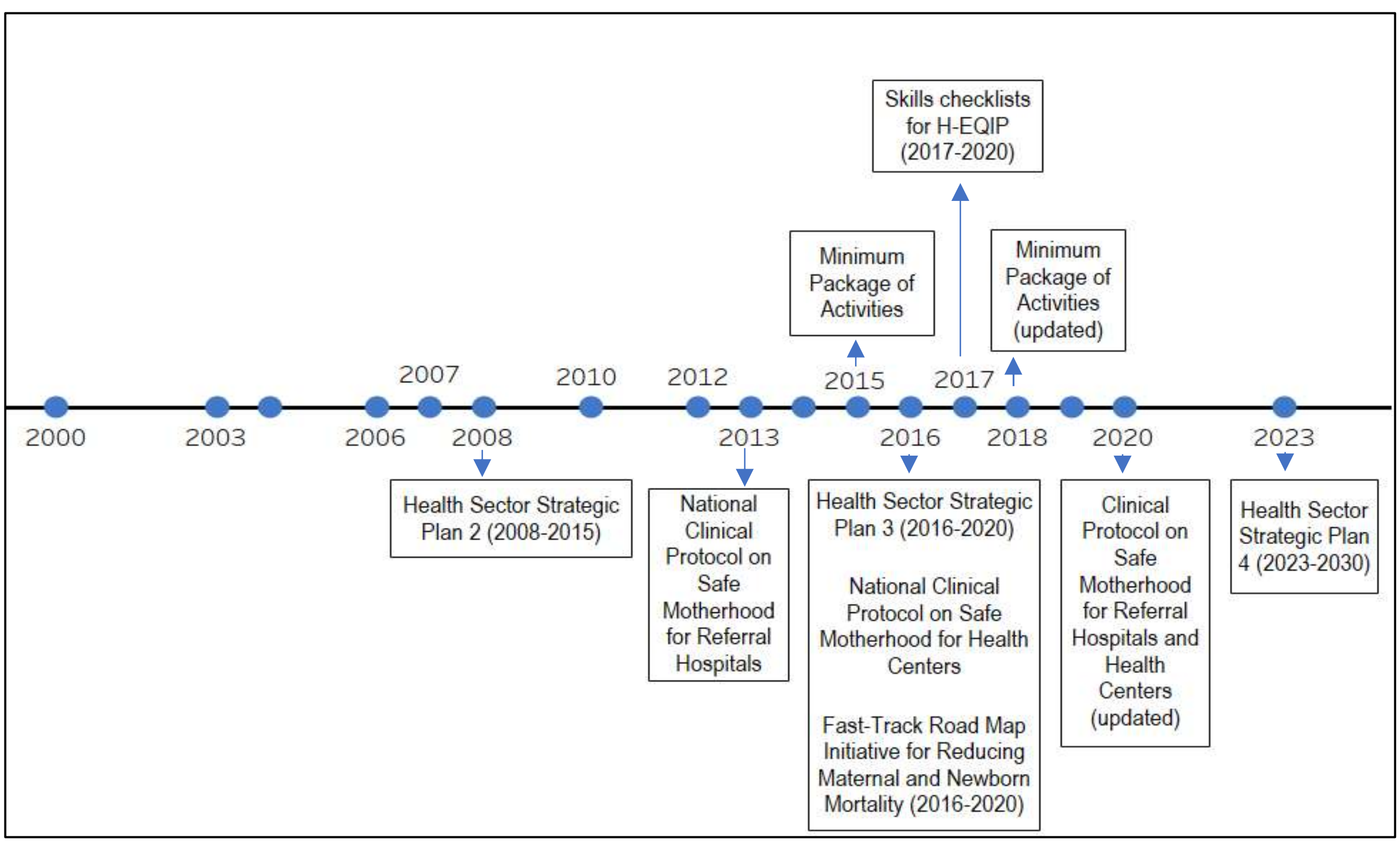

(c) Development of EENC program guidance, monitoring and evaluation

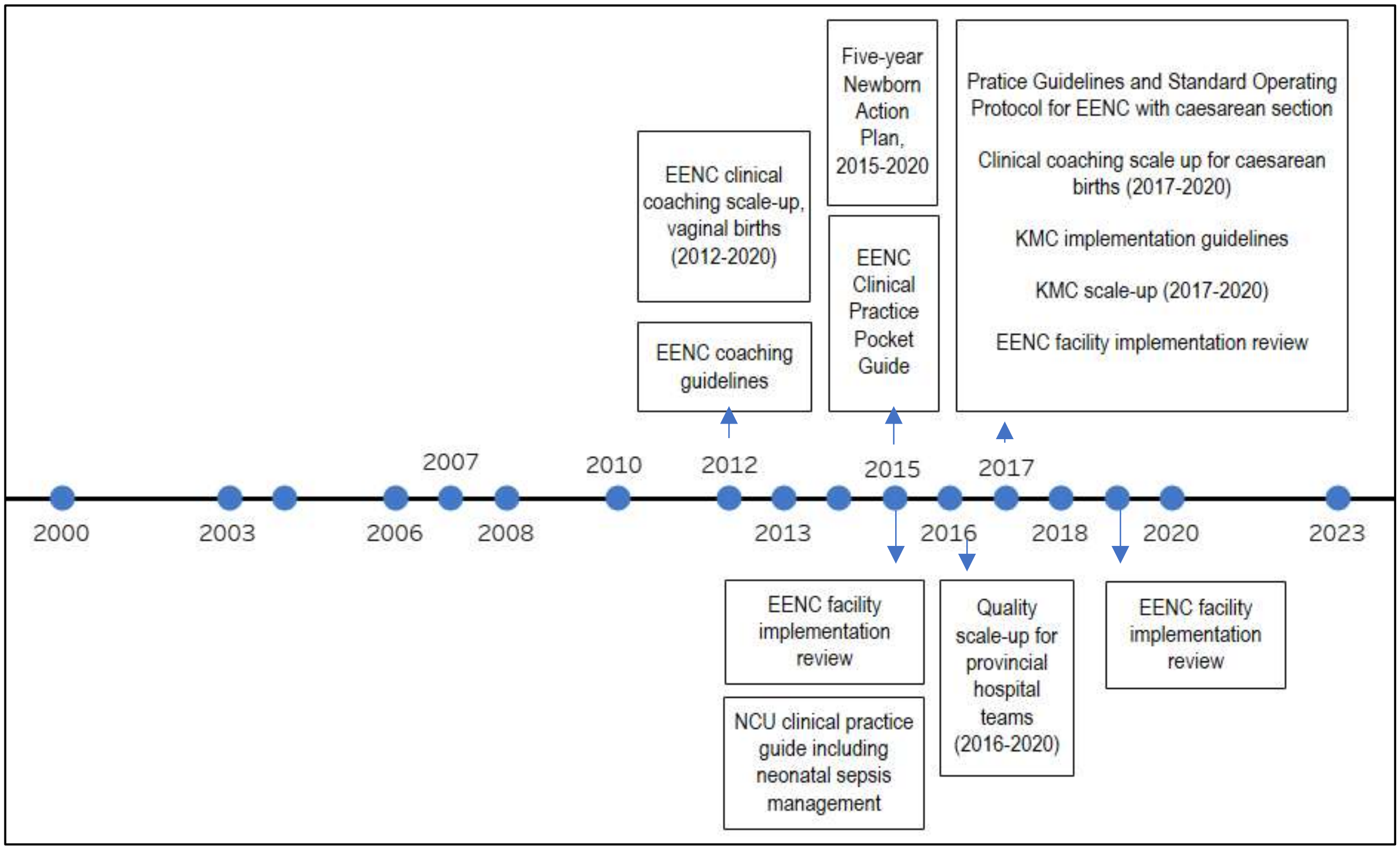

Supplement: Supplementary file 3 [file bmjoq-15-3-s003.pdf]
