# Supplementary material for: Contribution of improved quality of early essential newborn care to neonatal mortality decline in Cambodia, 2010–2022: a retrospective observational study
Source: BMJ Open Qual. 2026 Sep 1;15(3):e003920. doi: 10.1136/bmjoq-2025-003920 (PMC13536012; doi:10.1136/bmjoq-2025-003920)

Supplemental Figure 4: Neonatal mortality and birth practice trends, term and preterm/low birthweight newborns, Cambodia, 2011-2022

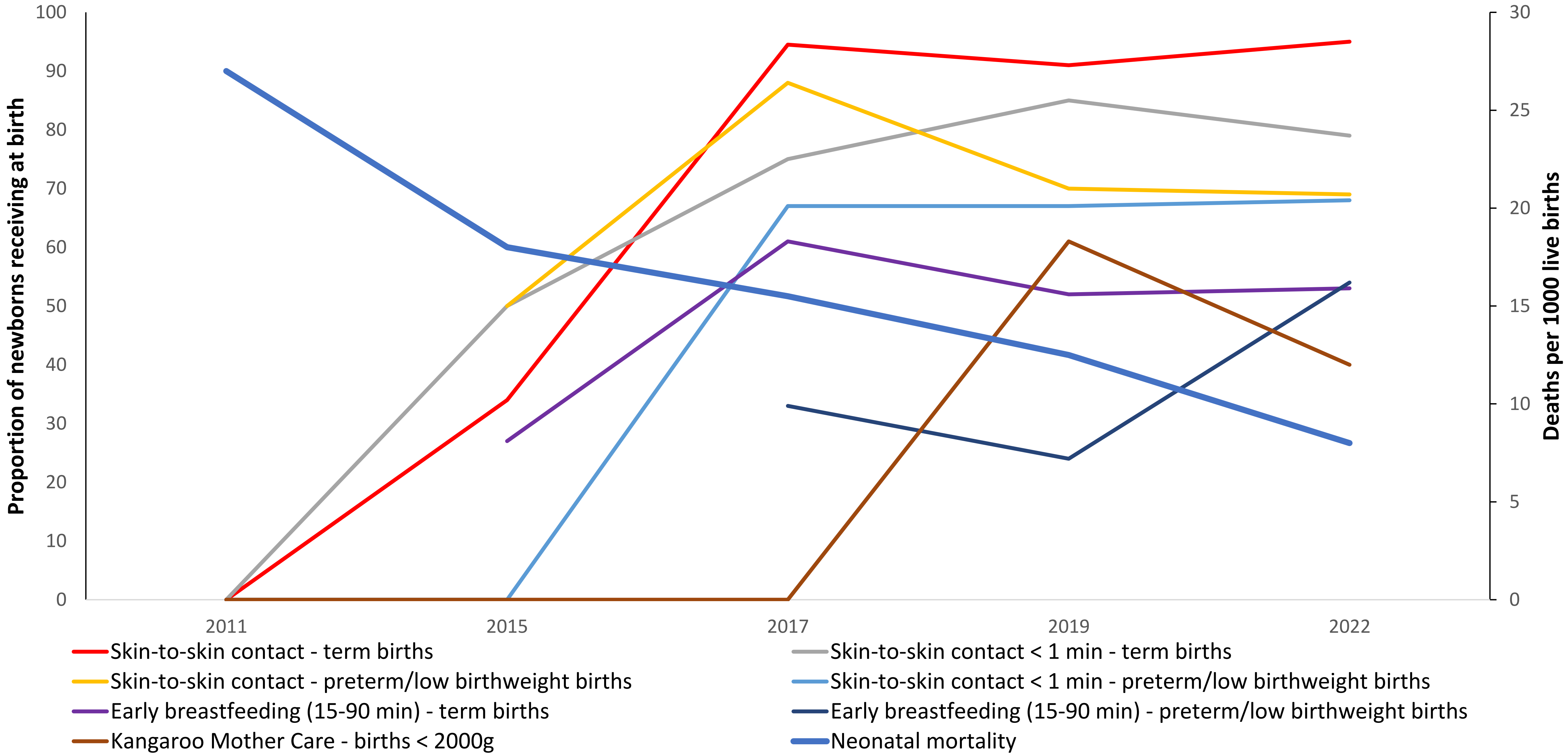

Supplement: Supplementary file 4 [file bmjoq-15-3-s004.pdf]
