# Supplementary material for: Contribution of improved quality of early essential newborn care to neonatal mortality decline in Cambodia, 2010–2022: a retrospective observational study
Source: BMJ Open Qual. 2026 Sep 1;15(3):e003920. doi: 10.1136/bmjoq-2025-003920 (PMC13536012; doi:10.1136/bmjoq-2025-003920)
Supplement: Supplementary file 5 [file bmjoq-15-3-s005.pdf]

Supplemental data

Supplemental Table 1: LiST assumptions: facility-based practice measures used in the model

Mean estimates

Indicator	Facility data used	Estimate practice quality	Estimated EENC facility coverage	Adjusted practice coverage
Clean birth environment	Q 1-9 observation checklist (EENC AIR Checklist 3.1)	2011 – 0%<br>2015 – 264/288 (91.7)<br>2017 -76/99 (76.7)<br>2019 – 157/234 (67.1)<br>2022 – 159/207 (76.8)	2011- 0<br>2015 -0.91 x 91.7<br>2017 -0.89 x 76.7<br>2019 – 0.89 x 67.1<br>2022 – 0.88 x 76.8	0%<br>83.5%<br>68.3%<br>59.7%<br>67.5%
Immediate drying and additional stimulation	Q 11, 12, 13 (EENC AIR Checklist 3.1)	2011 – 0%<br>2015 -70/96 (72.9)<br>2017 – 24/33 (72.7)<br>2019 – 59/78 (75.6)<br>2022 -51/69 (73.9)	2011 – 0<br>2015 -0.91 x 72.9<br>2017 – 0.89 x 72.7<br>2019 – 0.89 x 75.6<br>2022 – 0.88 x 73.9	0%<br>66.3%<br>64.7%<br>67.3%<br>65.0%
Thermal protection	Maternal exit interview – SSC received – 0-59m, 60-89m and ≥ 90m (EENC AIR Checklist 1.1)	2011 – 5%<br>2015 -15/18 (83.3)<br>2017 – 151/164 (92.1)<br>2019 -235/260 (91.9)<br>2022 – 267/290 (92.1)	2011 – 5<br>2015 – 0.91 x 83.3<br>2017 – 0.89 x 92.1<br>2019 – 0.89 x 91.9<br>2022 – 0.88 x 92.1	5%<br>75.8%<br>82.0%<br>81.8%<br>81.0%
Delayed cord clamping	Q 20, 21 (EENC AIR Checklist 3.1)	2011 – 1/20 (5.0)<br>2015 – 62/64 (96.9)<br>2017 -20/22 (90.9)<br>2019 -40/52 (76.9)<br>2022 -40/46 (87)	2011 – 5.0<br>2015 – 0.91 x 96.9<br>2017 – 89 x 90.9<br>2019 – 0.89 x 76.9<br>2022 – 0.88 x 87	5.0%<br>88.2%<br>80.9%<br>68.4%<br>76.6%
Clean cord care	Q 2, 7, 8, 9, 19 (EENC AIR Checklist 3.1)	2011 – 0<br>2015 -145/160 (90.6)<br>2017 – 45/55 (81.8)<br>2019 – 104/130 (80.0)<br>2022 -92/115 (80.0)	2011 – 0<br>2015 – 0.91 x 90.6<br>2017 – 0.89 x 81.8<br>2019 – 0.89 x 80.0<br>2022- 0.88 x 80.0	0%<br>82.5%<br>72.8%<br>71.2%<br>70.4%
Resuscitation	2014 RACHA supervisory FU data after first coaching – 2-3 hospitals	1699/1879 -90.4	2011- 5<br>2015 – 0.91 x 90.4<br>2017 – 0.89 x 90.4<br>2019 – 0.89 x 90.4<br>2022- 0.88 x 90.4	5 %<br>82.3%<br>80.5%<br>80.5%<br>79.6%
Management of newborn sepsis	No practice data available. Revised NN sepsis guidelines	LiST estimates	2011- 26.3<br>2015 –45.7<br>2017 –47.9<br>2019 –50.1	26.3%<br>45.7%<br>47.9%<br>50.1%

	introduced in 2017 – assume improvements followed		2022 – 52.3	52.3%
Preterm steroids	Maternal exit interview<br>24-34-week GA receiving corticosteroids (EENC AIR Checklist 1.1)	2011 – 0<br>2015 – 0<br>2017 – 2/7 (28.6)<br>2019 – 12/14 (85.7)<br>2022 – 9/12 (75%)	2011 – 0<br>2015 – 0<br>2017 – 0.89 x 28.6<br>2019 – 0.89 x 85.7<br>2022 – 0.88 x 75	0%<br>0%<br>25.5%<br>76.3%<br>66.0%
Preterm KMC	Maternal exit interview<br>Any KMC (EENC AIR Checklist 1.1)	2011- 0<br>2015 -0<br>2017 -0<br>2019 – 11/18 (61.1)<br>2022 – 4/10 (40)	2011-0<br>2015-0<br>2017 – 0<br>2019 – 0.89 x 61.1<br>2022 – 0.88 x 40	0%<br>0%<br>0%<br>54.4%<br>35.2%
Early breastfeeding 15-90	2010-DHS BF0-1m<br>Maternal exit interview (EENC AIR Checklist 1.1)	2011- 65.7<br>2015 – 4/15 (26.7)<br>2017 – 97/164 (59.1)<br>2019 – 128/260 (49.2)<br>2022 – 155/290 (53.4)	2011 – 65.7<br>2015 – 0.91 x 26.7<br>2017 – 0.89 x 59.1<br>2019 – 0.89 x 49.2<br>2022 – 0.88 x 53.4	65.7<br>24.3%<br>52.6%<br>43.8%<br>47.0%
Exclusive BF at discharge	2010- DHS EF 0-1 month – pre-EENC<br>Maternal exit interview (EENC AIR Checklist 1.1)	2011 – 73.4<br>2015 – 14/18 (77.8)<br>2017 – 141/164 (86.0)<br>2019 – 212/260 (81.5)<br>2022 – 266/290 (91.7)	2011 – 73.4<br>2015 – 0.91 x 77.8<br>2017 – 0.89 x 86<br>2019 – 0.89 x 81.5<br>2022 – 0.88 x 91.7	73.4%<br>70.8%<br>76.5%<br>72.5%<br>80.7%

Supplemental Table 2: LiST assumptions: facility-based practice measures used in the model

Lower bound estimates

Indicator	Facility data used	Estimate practice quality	Estimated EENC facility coverage	Adjusted practice coverage
Clean birth environment	Q 1-9 observation checklist (EENC AIR Checklist 3.1)	2011 – 0%<br>2015 – 224/288 (77.9)<br>2017 -48/99 (49.5)<br>2019 – 113/234 (48.3)<br>2022 – 118/207 (57.0)	2011- 0<br>2015 -0.91 x 77.9<br>2017 -0.89 x 49.5<br>2019 – 0.89 x 48.3<br>2022 – 0.88 x 57.0	0%<br>70.9%<br>44.1%<br>43.0%<br>50.2%
Immediate drying and additional stimulation	Q 11, 12, 13 (EENC AIR Checklist 3.1)	2011 – 1.8%<br>2015 -55/96 (57.4)<br>2017 – 15/33 (45.5)<br>2019 – 53/78 (68.0)<br>2022 -38/69 (55.1)	2011 –1.8<br>2015 -0.91 x 57.4<br>2017 – 0.89 x 45.5<br>2019 – 0.89 x 68.0<br>2022 – 0.88 x 55.1	1.8%<br>52.2%<br>40.5%<br>60.5%<br>48.5%
Thermal protection	Maternal exit interview – SSC received – 0-59m, 60-89m and ≥ 90m (EENC AIR Checklist 1.1)	2011 – 0%<br>2015 -11/18 (61.1)<br>2017 – 142/164 (87.2)<br>2019 -224/260 (86.2)<br>2022 – 256/290 (88.3)	2011 – 0<br>2015 – 0.91 x 61.1<br>2017 – 0.89 x 87.2<br>2019 – 0.89 x 86.2<br>2022 – 0.88 x 88.3	0%<br>55.6%<br>77.6%<br>76.7%<br>77.7%
Delayed cord clamping	Q 20, 21 (EENC AIR Checklist 3.1)	2011 – 1/20 (2.5)<br>2015 – 54/64 (84.4)<br>2017 -14/22 (63.6)<br>2019 -31/52 (59.6)<br>2022 -32/46 (69.6)	2011 – 2.5<br>2015 – 0.91 x 84.4<br>2017 – 89 x 63.6<br>2019 – 0.89 x 59.6<br>2022 – 0.88 x 69.6	2.5%<br>76.8%<br>56.6%<br>53.0%<br>61.2%
Clean cord care	Q 2, 7, 8, 9, 19 (EENC AIR Checklist 3.1)	2011 – 0<br>2015 -122/160 (76.3)<br>2017 – 30/55 (55.0)<br>2019 – 80/130 (61.5)<br>2022 -70/115 (60.9)	2011 – 0<br>2015 – 0.91 x 76.3<br>2017 – 0.89 x 55.0<br>2019 – 0.89 x 61.5<br>2022- 0.88 x 60.9	0%<br>69.4%<br>49.0%<br>54.7%<br>53.6%
Resuscitation	2014 RACHA supervisory FU data after first coaching – 2-3 hospitals	1588/1879 (85%)	2011- 2.5<br>2015 – 0.91 x 85`<br>2017 – 0.89 x 85<br>2019 – 0.89 x 85<br>2022- 0.88 x 85	2.5 %<br>77.4%<br>75.7%<br>75.7%<br>74.8%
Management of newborn sepsis	No practice data available. Revised NN sepsis guidelines	 <br>25%<br>33%	2011- 19.7<br>2015 –34.3<br>2017 –35.9<br>2019 –37.6	19.7%<br>34.3%<br>35.9%<br>37.6%

	introduced in 2017 – assume improvements followed		2022 – 39.2	39.2%
Preterm steroids	Maternal exit interview 24-34-week GA receiving corticosteroids (EENC AIR Checklist 1.1)	2011 – 0<br>2015 – 0<br>2017 – 8%<br>2019 – 60%<br>2022 – 47%	2011 – 0<br>2015 – 0<br>2017 – 0.89 x 8<br>2019 – 0.89 x 60<br>2022 – 0.88 x 47	0%<br>0%<br>7.1%<br>53.4%<br>41.4%
Preterm KMC	Maternal exit interview Any KMC (EENC AIR Checklist 1.1)	2011- 0<br>2015 -0<br>2017 -0<br>2019 – 39%<br>2022 – 17%	2011-0<br>2015-0<br>2017 – 0<br>2019 – 0.89 x 39<br>2022 – 0.88 x 17	0%<br>0%<br>0%<br>34.7%<br>15.0%
Early breastfeeding 15-90	2010- DHS EB -6 m – pre-EENC Maternal exit interview (EENC AIR Checklist 1.1)	2011- 64.0<br>2015 – 11%<br>2017 – 52%<br>2019 – 43%<br>2022 – 48%	2011 – 64.0<br>2015 – 0.91 x 11<br>2017 – 0.89 x 52<br>2019 – 0.89 x 43<br>2022 – 0.88 x 48	64.0%<br>10.0%<br>46.3%<br>38.3%<br>42.2%
Exclusive BF at discharge	2010- DHS EF 0-1 month – pre-EENC Maternal exit interview (EENC AIR Checklist 1.1)	2011 – 70%<br>2015 – 55%<br>2017 – 80%<br>2019 – 76%<br>2022 – 88%	2011 – 70.0<br>2015 – 0.91 x 55<br>2017 – 0.89 x 80<br>2019 – 0.89 x 76<br>2022 – 0.88 x 88	70.0%<br>50.1%<br>71.2%<br>67.6%<br>77.4%

Supplemental Table 3: LiST assumptions: facility-based practice measures used in the model

Upper bound estimates

**Indicator**	**Facility data used**	**Estimate practice quality**	**Estimated EENC facility coverage**	**Adjusted practice coverage**
Clean birth environment	Q 1-9 observation checklist (EENC AIR Checklist 3.1)	2011 – 27.8%<br>2015 – 277/288 (96.2)<br>2017 -89/99 (89.9)<br>2019 – 189/234 (80.8)<br>2022 – 185/207 (89.4)	2011- 27.8<br>2015 -0.91 x 96.2<br>2017 -0.89 x 89.9<br>2019 – 0.89 x 80.8<br>2022 – 0.88 x 89.4	27.8%<br>87.5%<br>80.0%<br>71.9%<br>78.7%
Immediate drying and additional stimulation	Q 11, 12, 13 (EENC AIR Checklist 3.1)	2011 – 27.8%<br>2015 -81/96 (84.4)<br>2017 – 29/33 (87.9)<br>2019 – 67/78 (85.9)<br>2022 -59/69 (85.5)	2011 – 27.8<br>2015 -0.91 x 84.4<br>2017 – 0.89 x 87.9<br>2019 – 0.89 x 85.9<br>2022 – 0.88 x 85.5	27.8%<br>76.8%<br>78.2%<br>76.5%<br>75.2%
Thermal protection	Maternal exit interview – SSC received – 0-59m, 60-89m and ≥ 90m (EENC AIR Checklist 1.1)	2011 – 27.8%<br>2015 -17/18 (94.2)<br>2017 – 156/164 (95.3)<br>2019 -243/260 (93.4)<br>2022 – 275/290 (94.7)	2011 – 27.8<br>2015 – 0.91 x 94.2<br>2017 – 0.89 x 95.3<br>2019 – 0.89 x 93.4<br>2022 – 0.88 x 94.7	27.8%<br>85.7%<br>84.8%<br>83.1%<br>83.3%
Delayed cord clamping	Q 20, 21 (EENC AIR Checklist 3.1)	2011 – 7/20 (34.1)<br>2015 – 64/64 (100)<br>2017 -21/22 (95.5)<br>2019 -45/52 (86.5)<br>2022 -43/46 (93.5)	2011 – 34.1<br>2015 – 0.91 x 100<br>2017 – 89 x 90.9<br>2019 – 0.89 x 86.5<br>2022 – 0.88 x 93.5	34.1%<br>91.0%<br>85.0%<br>76.9%<br>82.3%
Clean cord care	Q 2, 7, 8, 9, 19 (EENC AIR Checklist 3.1)	2011 – 27.8<br>2015 -153/160 (95.6)<br>2017 – 52/55 (95.5)<br>2019 – 117/130 (90.0)<br>2022 -104/115 (90.4)	2011 – 27.8<br>2015 – 0.91 x 95.6<br>2017 – 0.89 x 95`.5<br>2019 – 0.89 x 90.0<br>2022- 0.88 x 80.0	27.8%<br>87.0%<br>85.0%<br>80.1%<br>79.6%
Resuscitation	2014 RACHA supervisory FU data after first coaching – 2-3 hospitals	1761/1879 (94%)	2011- 34.1<br>2015 – 0.91 x 94<br>2017 – 0.89 x 94<br>2019 – 0.89 x 94<br>2022- 0.88 x 94	34.1 %<br>85.5%<br>83.78%<br>83.7%<br>82.7%
Management of newborn sepsis	No practice data available. Revised NN sepsis	50-70% of babies born in EENC implementing	2011- 32.9<br>2015 –57.1<br>2017 –59.9	32.9%<br>57.1%<br>59.9%

	guidelines introduced in 2017 – assume improvements followed	hospitals treated after 2017	2019 –62.6<br>2022 – 65.4	62.6%<br>65.4%
Preterm steroids	Maternal exit interview<br>24-34-week GA receiving corticosteroids (EENC AIR Checklist 1.1)	2011 – 0<br>2015 – 0<br>2017 – 5/7 (64.1)<br>2019 – 13/14 (96.0)<br>2022 – 1/12 (91.1%)	2011 – 0<br>2015 – 0<br>2017 – 0.89 x 64.1<br>2019 – 0.89 x 96.0<br>2022 – 0.88 x 91.1	0%<br>0%<br>57.1%<br>85.44%<br>80.2%
Preterm KMC	Maternal exit interview<br>Any KMC (EENC AIR Checklist 1.1)	2011- 0<br>2015 -0<br>2017 -3/7 (42.9)<br>2019 – 14/18 (77.8)<br>2022 – 7/10 (70)	2011-0<br>2015-0<br>2017 – 0.89 x 42.9<br>2019 – 0.89 x 77.8<br>2022 – 0.88 x 70	0%<br>0%<br>38.2%<br>69.2%<br>61.6%
Early breastfeeding 15-90	Maternal exit interview (EENC AIR Checklist 1.1)	2011- 67.4<br>2015 – 8/15 (52)<br>2017 – 109/164 (66.4)<br>2019 – 241/260 (92.8)<br>2022 – 276/290 (95.2)	2011 – 67.4<br>2015 – 0.91 x 52.0<br>2017 – 0.89 x 66.4<br>2019 – 0.89 x 92.8<br>2022 – 0.88 x 95.2	67.4%<br>47.3%<br>59.1%<br>82.6%<br>83.8%
Exclusive BF at discharge	2011- DHS EF 0-1 month – pre-EENC<br>Maternal exit interview (EENC AIR Checklist 1.1)	2011 – 76.6<br>2015 – 16/18 (91.0)<br>2017 – 148/164 (90.5)<br>2019 – 223/260 (85.8)<br>2022 – 273/290 (94.1)	2011 – 76.6<br>2015 – 0.91 x 91.0<br>2017 – 0.89 x 90.5<br>2019 – 0.89 x 85.8<br>2022 – 0.88 x 94.1	76.6%<br>82.8%<br>80.6%<br>76.4%<br>82.8%

Supplemental Table 4: Health facilities introducing EENC and staff coached in implementing facilities, Cambodia, 2015-2022

EENC measure	2015 n/N (%)	2017 n/N (%)	2019 n/N (%)	2022 n/N (%)
Health Facilities introducing EENC				
Total	1134/1246 (91.0)	1134/1271 (89.2)	1184/1324 (89.4)	1186/1354 (87.6)
National	0/5 (0.0)	0/5 (0.0)	2/5 (40.0)	2/5 (40.0)
Provincial and District	99/100 (99.0)	99/102 (97.1)	107/114 (93.9)	109/116 (93.9)
Primary	1035/1141 (90.7)	1035/1164 (88.9)	1075/1205 (89.2)	1075/1233 (87.2)
Total staff coached in EENC				
Total	3932/5487 (71.7)	4057/5347 (75.9)	4216/5578 (75.6%)	4487/5849 (76.7%)
National[1]	0 (0.0)	0 (0.0)	254	341
Provincial and District	877/1097 (79.9)	877/1099 (79.8)	931/1205 (77.3)	1019/1293 (78.8)
Primary	2908/4243 (68.5)	2926/4248 (68.9)	3031/4373 (69.3)	3127/4469 (70.0)

[1] Denominator data not available
